# No country for old men: five prevalent stereotypes affecting the life of the elderly

**DOI:** 10.3325/cmj.2020.61.184

**Published:** 2020-04

**Authors:** Stjepan Orešković

**Affiliations:** Andrija Štampar School of Public Health, University of Zagreb School of Medicine, Zagreb, Croatia *soreskov@snz.hr*

“That is no country for old men*”* is the first line of William Butler Yeats’ poem “Sailing to Byzantium” (1). Although the poem was written in 1928, the line became famous only in 2007, when the Coen brothers used it as a title of their film. The poem is a passionate expression of grief for the vanished country of the young. Even more, it is a lament on the carelessness and disrespect of adolescents for the wisdom of the past. In his book *A Vision,* Yeats says: “I think that in early Byzantium, maybe never before or since in recorded history, religious, aesthetic, and practical life (was) one, that architect and artificers ... spoke to the multitude and the few alike.” (2).

In the 21st century, people are living longer than ever. The theory of demographic transition defines the process of population aging, which brought Europe's population to the stage of maturity, as an increase in the median age of the general population. This process is the consequence of a steady decline of fertility rates (the average number of children that a woman aged 15-49 would have over her childbearing years) in the developed world. The rates are reaching a historical low, falling below 1.0 in countries such as Singapore (0.83) and Macau (0.95). Croatia is also experiencing a rapidly decreasing fertility rate, which in 2017 was as low as 1.4 children per a fertile woman – one of the lowest rates in the world. On the opposite side of the population pyramid, we are experiencing a rising life expectancy. All this is resulting in an unprecedented growth of the number of the elderly, with all-encompassing economic, social, political, and health consequences. In 2050, there will be more than 2.1 billion people aged 60 years and older (3). The number of people aged 80 years or older, the “oldest old,” is growing at an even faster pace, and most of the growth is expected in developing countries (4). The European region was the first region to embark on the second demographic transition, already in the late 1970s. By 2050, there will be 434 million people in this age group worldwide (4). Global chronological aging is undeniable, even though the Oxford Institute of Population Aging (5) suggests that population aging will slow considerably in Europe and will most significantly affect Asia, especially as Asia will reach stage five (low birth rate and low death rate) in the second part of the 21st century (6).

Aging societies are no longer an interesting phenomenon but rather an inevitable fact. Historically in certain cultures older people were often viewed as a burden to society. According to Japanese mythological traditions, when a person reaches the age of 70 he or she must travel to snowy mountains to die from freezing and starvation. The practice, known as *ubasute*, was depicted in the film *The Ballad of Narayama* (7), directed by Shoehi Immamura, which won Palme d’Or at the Cannes Film Festival in 1983. However, the perception of old people differs across cultures and between rural and urban societies. In Croatian rural communities, old age was associated with wisdom, maturity, and social status, and the quality of care depended on the general opinions about an individual’s “morality and kindness” or “frugality and moroseness, selfishness and stubborn resistance to the modernization of life” (8). Such attitudes were “strongly expressed by the common people and the elderly themselves referring to their own sensibility to old age and death in general, including their status in the society and family” (8). The development of urban society, industrialization, and later on post-industrial society strongly changed the attitudes toward the elderly, establishing new sets of values, norms, and institutional standards (9).

When aging became a recognized trend, the overall society, and social sciences in particular, started to develop negative views of it. Ageism is the most socially “accepted” of all types of prejudice, including sexism, classism, nationalism, and even gender prejudice. Ageism could be defined as language regularly and persistently characterizing older people in negative terms. It is the “stereotype and prejudice leading the discrimination against people based on their age. Ageism marginalizes and excludes older people in their communities and that's the reason why it's a widespread practice that has harmful effects on the health of older adults” (10). In the wake of the SARS-Cov-2 epidemics, the social media teemed with hostile comments and messages. Especially disturbing has been the use of the term “boomer remover,” a horrible nickname for COVID-19, most often touted by teenagers self-identified as “Gen-Z” or “Zoomers.” The Google search for “boomer remover” returns 11 400 000 results, most of them referring to the higher mortality rate among Baby Boomers, ie, the people in the 56 to 74 age cohort (11).

Institutional ageism involves the inclusion of age stereotypes and prejudice into formal rules and procedures, affecting the interests of the elderly in the processes of goal establishment and priority setting. WHO published several documents, reports, and guidelines on aging and health, indicating that it is “ready to jettison ageist concepts and champion a more ethical approach” and target ageism as a problematic social and institutional practice. However, WHO itself is not immune to conceptual bias against the elderly (12). These attitudes lead to the marginalization of older people within our communities and negatively affect their health and well-being (10). The language we use in public communication, the media, and even within our families is too often full of misconceptions, negative attitudes, and assumptions about older people. WHO listed ten leading misconceptions on aging and health (there is no typical older person; diversity in older age is random; a small proportion of the elderly is care-dependent; the aging population will increase health care costs but not as much as expected; 70 is not yet a new 60; good health in older age is not just absence of disease; families are essential but cannot provide the care many older people need; expenditures on more aged people are an investment, not a cost; it's not all about genes; mandatory retirement ages do not create jobs for youth) (13).

The common analytical mistake is to use only chronological-age measures when assessing how age relates to social welfare, health status, professional roles, and general social productivity of the populations (14). When Frank W. Notestien in the early 1950s developed a more formal theory of demographic transition, the public and established social sciences often expressed concerns about the roles and contributions of the elderly to the economic growth and social equilibrium (15). He was the first to argue that “viewed as a whole, the problem of aging is no problem at all. It is only the pessimistic way of looking at a great triumph of civilization” (15). Later on, positive assessments became more frequent, starting from the ideas of Edward Rosset, a Polish demographer working in the USA. He was among the first social scientists to note that the “most rapid social and economic development was achieved by countries with a significant share of older people.” (16). There has been almost no acknowledgment of the substantial benefits of an aging society and no knowledge-based guidance how to facilitate the transition to a cohesive, productive, secure, and equitable aging society. Such society might not only function effectively but might also provide a context that helps the individuals to age successfully (17)

What kind of prejudices do we project to the aging populations, and what is the difference between social perception and self-perception of the elderly? We will describe a few most widespread prejudices that are negatively affecting the lives of both the elderly and younger generations. Ageism is fuelled by the myths of the elderly swallowing our scarce resources.

The first prejudice represents a typical social and psychological construct that, in the reality of social interactions, we are dealing with a “typical” older person. Regardless of standard prejudices in real-life circumstances, a seventy-year-old person may have physical and mental capacities similar to a thirty-year-old. Older people rated themselves as being 20% more youthful than their chronological age, and this rating increased with age (18). A difference in both mental and physical capacities should be seen as an opportunity to develop productive and objective roles for the citizens who are becoming the most numerous global population group.

The second prejudice or misunderstanding originates from the common economic terminology, suggesting that people over 65 are not productive. This preconception is deeply rooted in the ancient myths and beliefs that old people are a leisure group that considerably contributes to both the economic burden on “productive generations” and to the global burden of disease (19). “Moody's report suggests that the aging population—often apocalyptically referred to as “the grey horde” or “the silver tsunami”—will dampen global economic growth for two decades” (20). Probably the largest obstacle to the inclusion of the elderly in the productive process is the perception of their unfamiliarity with technology. More than half of EU citizens (53%) feel that the use of technology is a major obstacle for the social inclusion and economic functioning of the elderly (21).

The third, and maybe the most widespread, prejudice is that the elderly contribute to the increase in health care expenditure even more than technological advancements and price growth. In Croatia, risky health-related behavior among the elderly is widespread, resulting in 45% of the population 50+ being overweight and 27% suffering from obesity. One in three women aged 50+ have healthy body weight compared with one in five men, changing the pathology of the fast-aging society (22). Given that 20.45% of the population is already aged 65 years or older, the critical health challenge for Croatian policymakers is to improve the quality of the primary and secondary prevention services for non-communicable diseases and chronic illnesses (22). Although it is true that older people increase health care expenditure, recent research shows that the major generators of total health care expenditures growth are technological advancements and price growth. “While older people, on average, incur higher per person healthcare spending than younger people, the proportions of the population at the oldest and most costly age groups increase very slowly over time. In the European Union, we estimate the changing age-mix to result in the increase of the average annual growth in per person health spending by no more than 0.6 additional percentage points per year between 2015 and 2050” (23).

The fourth prejudice is the institutionalization of retirement, being at the same time the root cause and consequence of the exclusion of the elderly from the working environment. Policies and practices related to the retirement age vary considerably around the world. In Mexico, women retire at 75 years and in Slovakia at 54.5 years. Obviously, this has little to do with real working capacity, biological variations, or psychological setup. In most cases, formal retirement age is the result of the pressure from trade unions, fluctuation in employment opportunities, long-term policies for restructuring the economy or cultural values and influences. A positive example of policies that run counter to prejudiced, ignorant, and lackadaisical practices, and vested interests, can be found in Singapore. This country, a mixture of authoritarian state and democracy, has achieved a paramount change in labor participation rate, increasing the number of workers aged 65+ from 14.3% in 2006 to 26.8% in 2017 – a 53.3% growth in only eleven years. In South Korea, the percentage is even higher – 31.5% (24). Iceland tops the chart of the developed economies in terms of effective retirement age, which is 69.7 years (25). At the same time in Croatia, labor participation rate for citizens aged 65+ is 1%! In addition, the aging index, expressing the ratio between the group aged 65+ and the group aged under 15, in Croatia is more than 100 000. The ratio of workers per a retiree decreased from 2.7 in 1989 to 1.17 in 2011 (26).

The fifth prejudice is about knowing and telling the truth about aging and the elderly. The fundamental question is whether we can obtain reliable and credible information on the elderly more than 70 years after the establishment of demographic transition theory. Making decisions based on emotions, fears, prejudice, or best guesses rather than on evidence can lead to isolation, separation, or discrimination of the elderly and affect the population health and public policies.

WHO strongly emphasizes the need to improve the measurement, monitoring, and understanding of a wide range of aging issues, arguing for focused research and new metrics and analytical methods (27). The existing measures of overall mortality are biased toward the conditions that excessively kill older people. The term “premature mortality” justifies existing age discrimination in health care by implying that survival after the age of 70 is less important than survival at younger ages. The same applies to the concept of “years of potential life lost,” which is setting the arbitrary age threshold between 65 and 80, resulting in a zero value of burden of disease for deaths that occur in later life (28).

WHO has invested time and resources to improve health statistics and distribute information related to aging populations. The measures of population aging based on chronological age have become outdated, and alternative standards have been developed. Three most sensitive indexes are the Aging Society Index, Global Age-Watch Index, and Active Aging Index. The most comprehensive one, the Active Aging Index (29), was conceptualized by the United Nations Economic Commission for Europe. Its objective is to measure the older people’s ability to participate in social activities and live independent lives. In addition, the index measures the proportion of the elderly that participate in paid employment and active aging capacity. It comprises 22 separate indicators logically grouped into four domains. The Global Age-Watch focuses on objective measures of health functioning that are comparable across time and populations, including health systems in general and the characteristics of the health care organizations, financing mechanisms, workforce, policies, facilities, and medicine and equipment. Since millions of older people are being left without access to health services or denied their right to health, Global Age Watch (27) is monitoring the adoption of the Sustainable Development Goals and how it relates to providing “health for all at all ages.” The Aging Society Index (29) is a continuous measure of the societal adaptation based on the five domain scores weighted in the following order: 25% for well-being, 22% for productivity, 25% for equity, 19% for security, and 18% for social cohesion. The scores are further aggregated by researchers giving relative weights to the total score for socially adaptive aging. The country holding the highest score is Norway, followed by Sweden. Tools such as the Aging Society Index help identify countries that “may serve as models for improvement for countries with specific gaps” (30). Without an accurate and unbiased understanding of the facts, expectations, trends, and projections related to the elderly we cannot measure social progress. If we do not gain such an understanding, our society will continue to develop a profound paradox: the largest social age group, representing a significant majority of the population, the majority of voters and patients, will be continuously treated as a minority, separated and singled out from the others, experiencing unequal treatment and collective discrimination.

The contemporary attitude toward the elderly is clearly visible in the lack of urgent response to the SARS-CoV-2 epidemic by too many governments. Such response was probably related to the perception of the disease as a condition primarily lethal to old people “less worthy of the best efforts to contain it,” as WHO’s director-general has recently noted (31). Such social and political discrimination directly affects huge numbers of residents of nursing homes, the institutions that are frequently under-regulated and provide care of very questionable quality. It the era of the global spread of SARS-Cov-2, national health and social policies must consider the already horrendous living conditions of many older people. Particularly vulnerable to social distancing are nursing home residents and the elderly living alone who in quarantine conditions face barriers to obtaining food and essential supplies. We are already witnessing the deadly consequences of institutional ageism. Seventeen residents of Monte Hermoso nursing home in Madrid died because the institution dismissed their employees in the wake of the outbreaks (32).

While “sailing” to Byzantium, the early Christian city populated with young students frequenting Plato's Academy, Yeats took a spiritual journey to eternity. If he had lived longer, Yeats (who died in 1939) would have found himself in a world populated with the elderly, but a world with “no country for old men.”
